# Inflammatory proteins may mediate the causal relationship between gut microbiota and inflammatory bowel disease: A mediation and multivariable Mendelian randomization study

**DOI:** 10.1097/MD.0000000000038551

**Published:** 2024-06-21

**Authors:** Yu-Liang Huang, Jin-Min Zheng, Zheng-Yi Shi, Huan-Huan Chen, Xiao-Tong Wang, Fan-Biao Kong

**Affiliations:** a Guangxi Medical University, Nanning, Guangxi, People’s Republic of China; b Department of Colorectal and Anal Surgery, Guangxi Academy of Medical Sciences, People’s Hospital of Guangxi Zhuang Autonomous Region, Institute of Minimally Invasive Technology and Applications Guangxi Academy of Medical Sciences, Nanning, Guangxi Zhuang Autonomous Region, People’s Republic of China; c Departments of Gastrointestinal, Hernia and Enterofistula Surgery, People’s Hospital of Guangxi Zhuang Autonomous Region, Institute of Minimally Invasive Technology and Applications Guangxi Academy of Medical Sciences, Nanning, Guangxi Zhuang Autonomous Region, People’s Republic of China.

**Keywords:** Crohn’s disease, gut microbes, inflammatory proteins, Mendelian randomization, ulcerative colitis

## Abstract

This research investigates the causal relationships among gut microbiota, inflammatory proteins, and inflammatory bowel disease (IBD), including crohn disease (CD) and ulcerative colitis (UC), and identifies the role of inflammatory proteins as potential mediators. Our study analyzed gut microbiome data from 13,266 samples collected by the MiBioGen alliance, along with inflammatory protein data from recent research by Zhao et al, and genetic data on CD and UC from the International Inflammatory Bowel Disease Genetics Consortium (IIBDGC). We used Mendelian randomization (MR) to explore the associations, complemented by replication, meta-analysis, and multivariable MR techniques for enhanced accuracy and robustness. Our analysis employed several statistical methods, including inverse-variance weighting, MR-Egger, and the weighted median method, ensuring comprehensive and precise evaluation. After MR analysis, replication and meta-analysis, we revealed significant associations between 11 types of gut microbiota and 17 inflammatory proteins were associated with CD and UC. Mediator MR analysis and multivariable MR analysis showed that in CD, the CD40L receptor mediated the causal effect of *Defluviitaleaceae UCG-011* on CD (mediation ratio 8.3%), and the Hepatocyte growth factor mediated the causal effect of *Odoribacter* on CD (mediation ratio 18%). In UC, the C-C motif chemokine 4 mediated the causal effect of *Ruminococcus2* on UC (mediation ratio 4%). This research demonstrates the interactions between specific gut microbiota, inflammatory proteins, and CD and UC. Furthermore, the CD40L receptor may mediate the relationship between *Defluviitaleaceae UCG-011* and CD; the Hepatocyte growth factor may mediate the relationship between *Odoribacter* and CD; and the C-C motif chemokine 4 may mediate the relationship between *Ruminococcus2* and UC. The identified associations and mediation effects offer insights into potential therapeutic approaches targeting the gut microbiome for managing CD and UC.

## 1. Introduction

Inflammatory bowel disease (IBD), encompassing crohn disease (CD) and ulcerative volitis (UC), is a type of chronic immunological illness. Its characteristics include persistent pain in the abdomen, frequent diarrhea, and episodes of bleeding from the rectum.^[1]^ In Western countries, the incidence of IBD has been exhibiting a consistent upward trend, with an annual growth rate of 2.86%. Estimations suggest a notable increase in the incidence of IBD by 2030.^[2]^ IBD patients may suffer from malnutrition, experiencing weight loss, and a heightened likelihood of developing colorectal cancer, which presents a substantial public health challenge, particularly within the older adults.^[3]^ Although the clinical features of IBD have been extensively studied, its complex etiology and pathogenesis have not been fully revealed.^[4]^ The interaction of genetic, environmental factors, immune responses, and gut microbiota is involved.^[5–7]^

In recent years, there has been a growing focus among researchers on exploring the relationship between the gut microbiota and IBD. This complex microbial ecosystem in the gastrointestinal tract is involved in several functions, including the metabolism of bile acids,^[8]^ production of short-chain fatty acids (SCFAs) and nutrient synthesis.^[9,10]^ Furthermore, it is instrumental in the regulation of the host immune response.^[11]^ Some clinical observational studies have identified certain harmful bacteria directly associated with the onset of IBD,^[12–14]^ while some potentially protective bacteria are usually found in reduced abundance in IBD.^[15]^ Therefore, the imbalance in the gut microbiota is considered as a critical element in the onset of IBD. Moreover, inflammatory proteins are essential in regulating the interaction between the gut microbiota and immune system.^[6,16]^ For example, the positive correlation between gastric carcinoma group and cytokines of TNF-α and IL-6, which mediate systemic immune responses, hinting at the microbiota potential role in influencing IBD progression through a series of inflammatory signal signals.^[17,18]^ Research on *Escherichia coli* further supports the regulatory role of inflammatory proteins in intestinal health.^[19,20]^ These findings suggest that inflammatory proteins may play a role in the development of the gut microbiota and IBD. However, the ability to establish causality is limited due to observational studies being susceptible to confounding variables and the problem of reverse causality.^[21,22]^ Thus, Mendelian randomization (MR) studies have been introduced to provide more reliable evidence of causality.

Although previous MR studies have identified a causal correlation between gut microbiota and IBD.^[23–25]^ However, it remains uncertain whether inflammatory proteins mediate the relationship between gut microbiota and the development of IBD. Based on that, this study specifically focuses on CD and UC, utilizing distinct datasets for CD and UC, we aim to explore whether inflammatory proteins serve as mediators in the relationship between gut microbiota and CD and UC through the MR approach. With this research design, we hope to reveal the specific role of inflammatory proteins in how gut microbiota influences CD and UC, thereby providing a scientific basis for developing targeted treatment strategies. This will broaden our comprehension of the gut microbiota impact on gut health and potentially find novel therapeutic strategies for managing CD and UC, especially for those patients experiencing limited effects from traditional treatments.

## 2. Methods

### 2.1. Study design

**Figure 1.**
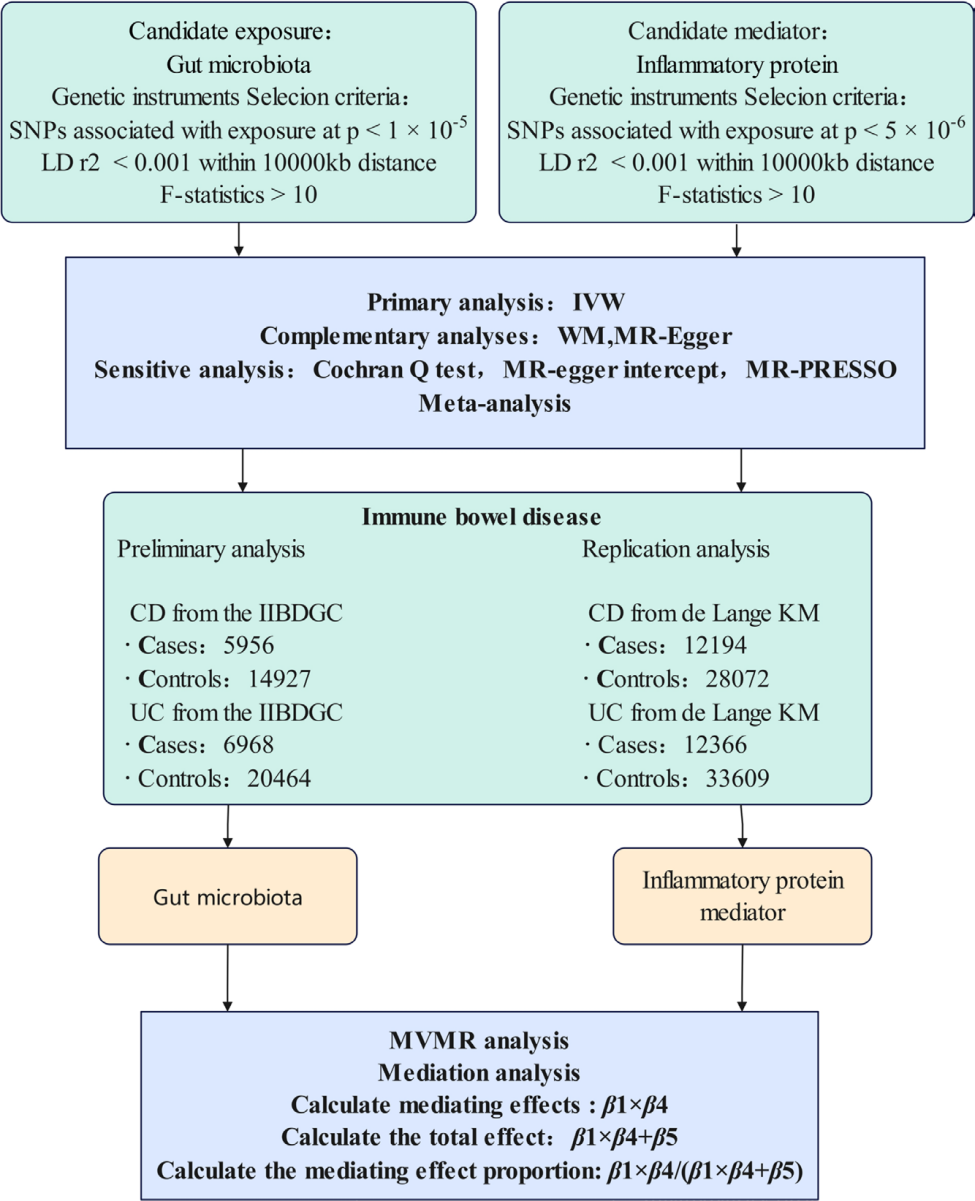
Mendelian randomization (MR) process diagram. CD = Crohn disease, IIBDGC = International Inflammatory Bowel Disease Genetics Consortium, IVW = inverse variance weighted, LD = linkage disequilibrium, MR-PRESSO = mendelian randomization pleiotropy residual sum and outlier, MVMR = multivariate mendelian randomization, SNPs = single nucleotide polymorphisms, UC = ulcerative colitis, WM = weighted median.

This MR study is divided into 2 phases (Fig. 1). In the first phase, we preliminarily analyzed the causal relationship between the gut microbiome, inflammatory proteins, and CD or UC, and then screened for candidate gut microbiomes and potential intermediary inflammatory proteins. In the second phase, we conducted multivariable MR analysis to examine whether, after adjusting for the mutual regulation between the gut microbiome and inflammatory proteins, a causal relationship with CD or UC still exists, and to determine whether the candidate inflammatory proteins serve as mediators between the gut microbiome and CD or UC, and calculate the proportion of mediation effect. Our MR analysis is based on the following 3 assumptions: The instrumental variables (IVs) are associated with the exposure; IVs are not related to any confounding factors; IVs only affect the outcome through exposure. Since genetic variations are formed randomly at conception according to Mendel laws, the outcomes of MR analysis tend to be minimally influenced by confounding factors. This study uses summary data from GWAS studies, mainly targeting research on Europeans by professional organizations.

### 2.2. Data sources

The gut microbiome data comes from the largest Genome-wide association studies (GWAS) dataset currently available from the MiBioGen consortium. This study encompasses 24 cohorts, totaling 18,340 individuals, and includes data on 131 genera. Our research will primarily investigate these genera.^[26]^ Data for 91 inflammatory protein plasma pQTLs comes from a recent study by Zhao et al, which conducted a genome-wide pQTL mapping and meta-analysis of 91 inflammatory proteins across 11 cohorts of 14,824 Europeans.^[27]^ Data on CD and UC comes from the International Inflammatory Bowel Disease Genetics Consortium (IIBDGC), incorporating 6968 UC patients and 20,464 controls, as well as 5956 CD patients and 14,927 controls for this study.^[28]^ Meta-data comes from the study by de Lange KM, which included 12,194 CD patients and 28,072 controls, 12,366 UC patients and 33,609 controls.^[29]^

### 2.3. IVs selection

We selected single nucleotide polymorphisms (SNPs) of the gut microbiome as IVs based on a *P* value threshold (<1 × 10^−5^), a commonly used threshold in previous gut microbiome studies. For selecting IVs for inflammatory proteins, we employed a *P* value threshold (<5 × 10^−6^). Linkage disequilibrium (LD) analysis was conducted using the European reference data from the 1000 Genomes Project. All these IVs were clustered within a ± 10000 kb distance with an R^2^ threshold of < 0.001 for LD. For palindromic SNPs, allele frequency information was used to identify the forward-strand alleles. Ultimately, F-statistics were calculated for all analyzed SNPs, with those having an F-statistic under 10 deemed weak instruments.^[30]^

### 2.4. MR analysis

To preliminarily explore the potential causal links between gut microbiota, inflammatory proteins, and inflammatory bowel disease initially, we utilized the inverse variance weighted (IVW) method. This approach combines individual SNP Wald ratios to evaluate outcomes. We also determined the *P* value for Cochrane Q to evaluate result heterogeneity. In cases of heterogeneity, we applied the IVW random effects model; otherwise, we opted for the fixed effects model. Sensitivity and supplementary analyses were performed using the MR-Egger and weighted median (WM) methods, respectively. Additionally, the Mendelian randomization pleiotropy residual sum and outlier (MR-PRESSO) method was used to identify and adjust for potential outliers, thereby refining the causal effect estimations.^[31]^

### 2.5. Replication and meta-analysis

To further validate the stability of the relationship between potential gut microbiota and inflammatory proteins discovered through the forward MR method, we replicated the IVW analysis on another dataset. Specifically, we initially conducted a preliminary analysis using data from the IIBDGC and replicated the analysis based on the study data of de Lange KM. By integrating the results of these 2 MR analyses, we ultimately confirmed specific gut microbiota and inflammatory proteins that have a causal relationship with CD and UC. During the meta-analysis, we selected the appropriate model based on the results of the IVW: a random-effects model in the face of result heterogeneity, and a fixed-effects model when the results were consistent.

### 2.6. MVMR and mediation analysis

**Figure 2.**
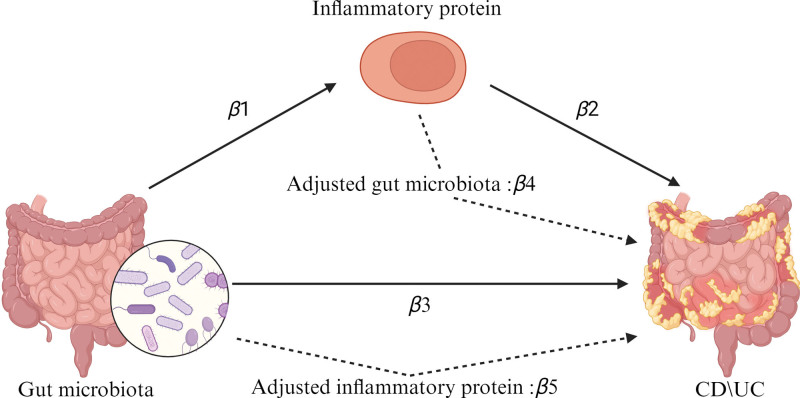
MVMR and mediation effect calculation mediation effect proportion = β1β4/ (β1β4 + β5). MVMR = multivariable Medelian randomization.

We first calculated the β1 values from the gut microbiota to the inflammatory proteins using 2-sample MR, then determined the β2 values from the inflammatory proteins to CD, and UC, we finally computed the β3 values from the gut microbiota to both CD and UC. Subsequently, we used multivariable MR (MVMR) to ascertain the causal relationship between the remaining exposures and CD, UC after adjusting for either gut microbiota or inflammatory proteins. After mutual adjustment, the β values for inflammatory proteins to CD, UC were designated as β4, and those for gut microbiota to CD, UC were designated as β5. The mediation proportion of inflammatory proteins was calculated using the formula “mediation effect proportion = β1β4/ (β1β4 + β5),” where β1β4 represents the mediating effect in the mechanism, β5 represents the direct effect in the mechanism, and β1β4 + β5 represents the total effect of the exposure in the mechanism (Fig. 2).

## 3. Results

In the GWAS data for gut microbiota, inflammatory proteins, and CD and UC, the SNPs used for MR analysis are listed in Supplementary Tables 1, http://links.lww.com/MD/M896 and 2, http://links.lww.com/MD/M897. For all SNPs utilized in the analysis, the F-statistics exceeded 10.

### 3.1. The causal relationship between gut microbiota and CD, UC

**Figure 3.**
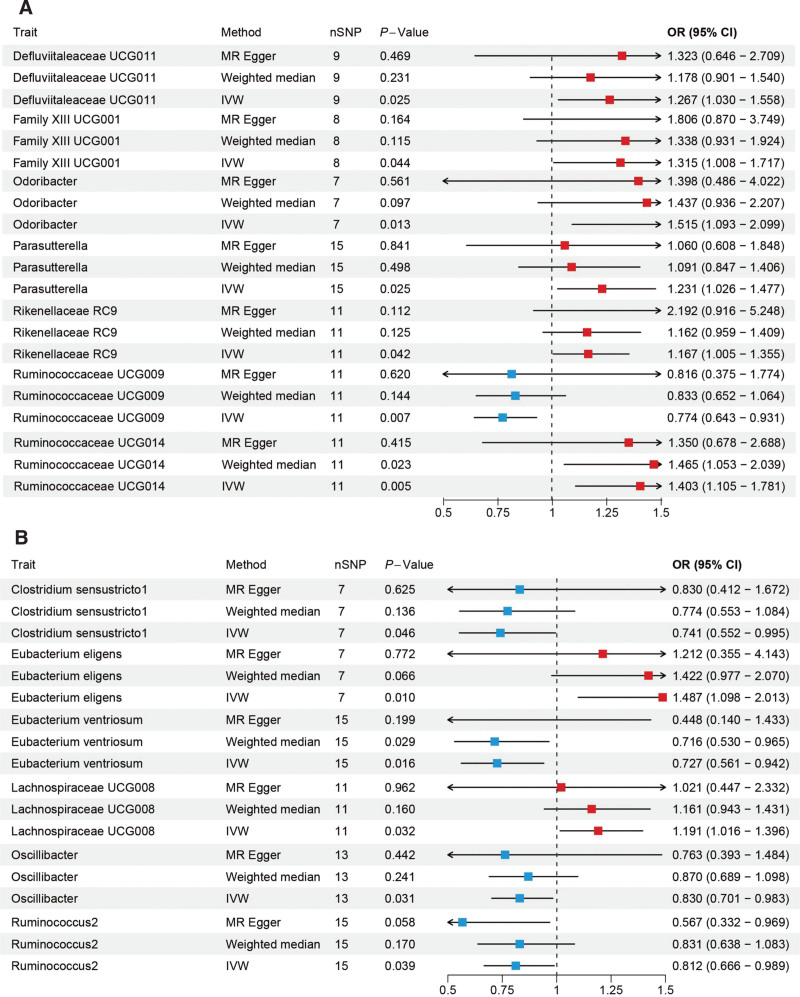
(A) MR analysis of the causal relationship between the gut microbiota and CD. (B) MR analysis of the causal relationship between the gut microbiota and UC. CD = Crohn disease, MR = Mendelian randomization, UC = ulcerative colitis.

Using IVW as the primary analysis method, and with the β-value results of IVW, MR-Egger, and WM analyses pointing in the same direction, and no pleiotropy existing after MR-PRESSO analysis (Supplementary Tables 3, http://links.lww.com/MD/M898 and 4, http://links.lww.com/MD/M899), we conclude that in CD, an increase in *Ruminococcaceae UCG014* (OR = 1.40, 95%CI = 1.10–1.78, *P* = .005), *Odoribacter* (OR = 1.51, 95%CI = 1.09–2.09, *P* = .012), *Defluviitaleaceae UCG011* (OR = 1.26, 95%CI = 1.03–1.55, *P* = .025), *Parasutterella* (OR = 1.23, 95%CI = 1.02–1.47, *P* = .025), *Rikenellaceae RC9* (OR = 1.16, 95%CI = 1.00–1.35, *P* = .042), and *Family XIII UCG001* (OR = 1.31, 95%CI = 1.00–1.71, *P* = .043) may increase the risk of CD, while an increase in *Ruminococcaceae UCG009* (OR = 0.77, 95%CI = 0.64–0.93, *P* = .006) may decrease the risk of CD (Fig. 3A).

In UC, an increase in *Eubacterium eligens group* (OR = 1.48, 95%CI = 1.09–2.01, *P* = .010), *Lachnospiraceae UCG008* (OR = 1.19, 95%CI = 1.01–1.39, *P* = .031) may increase the risk of UC, while an increase in *Eubacterium ventriosum group* (OR = 0.72, 95%CI = 0.56–0.94, *P* = .015), *Oscillibacter* (OR = 0.83, 95%CI = 0.70–0.98, *P* = .031), *Ruminococcus 2* (OR = 0.81, 95%CI = 0.66–0.98, *P* = .038), and *Clostridium sensu stricto 1* (OR = 0.74, 95%CI = 0.55–0.99, *P* = .046) may decrease the risk of UC (Fig. 3B).

### 3.2. The causal relationship between inflammatory proteins and CD, UC

Using IVW as the main analysis method, with the β-value results of IVW, MR-Egger, and WM analyses pointing in the same direction, and no pleiotropy detected after MR-PRESSO analysis (Supplementary Tables 5, http://links.lww.com/MD/M900 and 6, http://links.lww.com/MD/M901).

**Figure 4.**
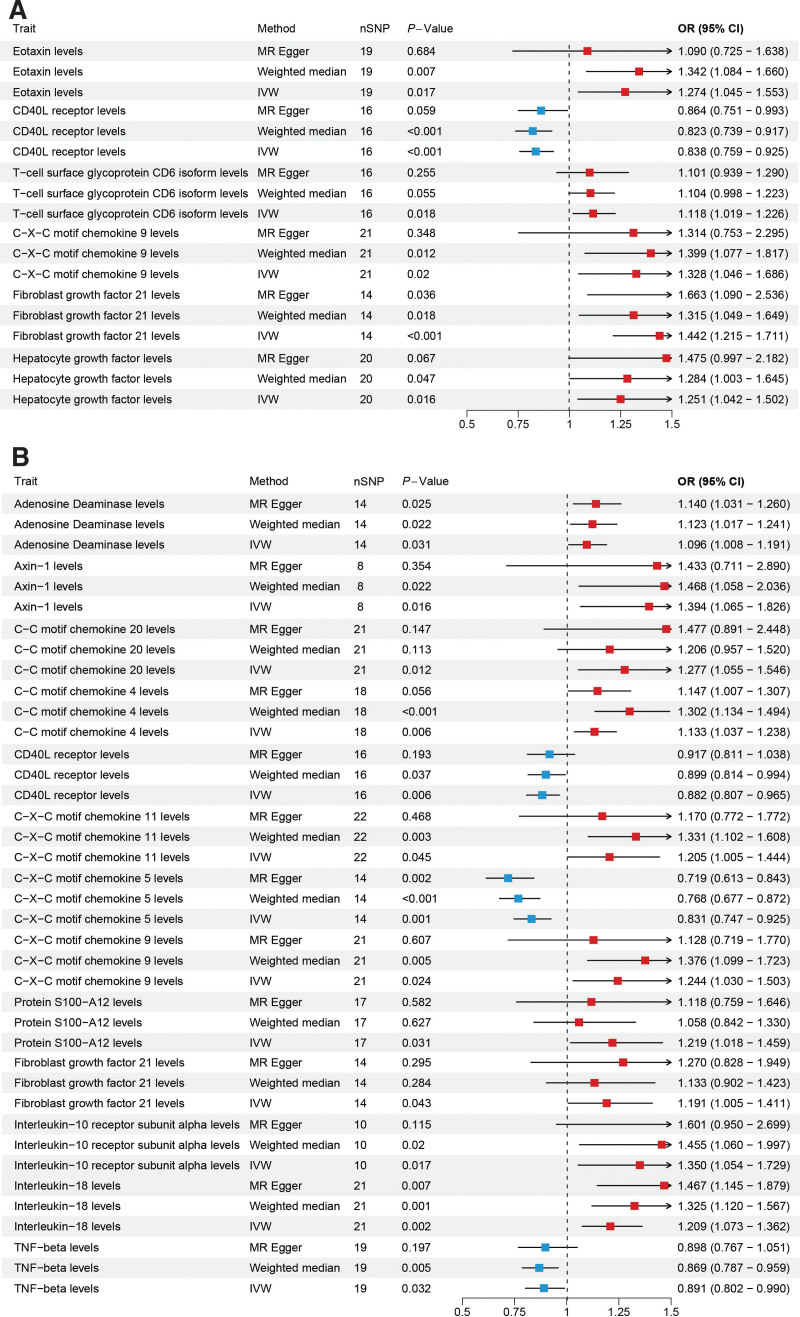
(A) MR analysis of the causal relationship between the inflammatory proteins and CD. (B) MR analysis of the causal relationship between the inflammatory proteins and UC. CD = Crohn disease, MR = Mendelian randomization, UC = ulcerative colitis.

In CD, an increase in Fibroblast growth factor 21 (OR = 1.44, 95%CI = 1.21–1.71, *P* < .001), Hepatocyte growth factor(HGF) (OR = 1.25, 95%CI = 1.04–1.50, *P* = .016), Eotaxin (OR = 1.27, 95%CI = 1.04–1.55, *P* = .016), T-cell surface glycoprotein CD6 isoform (OR = 1.11, 95%CI = 1.01–1.22, *P* = .018), and C-X-C motif chemokine 9 (CXCL9) (OR = 1.32, 95%CI = 1.04–1.68, *P* = .019) may increase the risk of CD, while an increase in CD40L receptor (OR = 0.83, 95%CI = 0.75–0.92, *P* < .001) may reduce the risk of CD (Fig. 4A).

In UC, an increase in C-C motif chemokine 20 (CCL20) (OR = 1.27, 95%CI = 1.05–1.54, *P* = .011), Axin-1 (OR = 1.39, 95%CI = 1.06–1.82, *P* = .015), Interleukin-10 receptor subunit alpha (IL-10RA) (OR = 1.34, 95%CI = 1.05–1.72, *P* = .017), C-X-C motif chemokine 9 (OR = 1.24, 95%CI = 1.02–1.50, *P* = .023), Adenosine Deaminase (OR = 1.09, 95%CI = 1.00–1.19, *P* = .031), Protein S100-A12 (OR = 1.21, 95%CI = 1.01–1.45, *P* = .031), Interleukin-18 (IL-18) (OR = 1.20, 95%CI = 1.07–1.36, *P* = .001), C-C motif chemokine 4 (CCL4) (OR = 1.13, 95%CI = 1.03–1.23, *P* = .005), Fibroblast growth factor 21 (FGF21) (OR = 1.19, 95%CI = 1.00–1.41, *P* = .043), and C-X-C motif chemokine 11 (CXCL11) (OR = 1.20, 95%CI = 1.00–1.44, *P* = .044) may increase the risk of UC. Conversely, an increase in CD40L receptor (OR = 0.88, 95%CI = 0.80–0.96, *P* = .006), TNF-beta (OR = 0.89, 95%CI = 0.80–0.99, *P* = .031), and C-X-C motif chemokine 5 (OR = 0.83, 95%CI = 0.74–0.92, *P* < .001) may reduce the risk of UC (Fig. 4B).

### 3.3. Results of meta-analysis

To enhance the robustness of our selected results, we conducted a meta-analysis on all outcomes. The analysis revealed that in CD, the results for gut microbiota *Rikenellaceae RC9* and *Family XIII UCG001* were not significant (*P* > .05) (Supplementary Figure 1, http://links.lww.com/MD/M894). Similarly, in UC, the results for inflammatory proteins Adenosine Deaminase and Protein S100-A12 were not significant (*P* > .05). These results were not included in subsequent analyses (Supplementary Figure 2, http://links.lww.com/MD/M895)

### 3.4. Potential mediating inflammatory proteins

MR analyzed the causal relationships between selected gut microbiota and inflammatory proteins.

In CD, *Defluviitaleaceae UCG011* was negatively correlated with C-X-C Motif Chemokine 9 (β = −0.15, *P* = .023) and the CD40L Receptor (β = −0.11, *P* = .025), while *Odoribacter* showed a positive correlation with T-cell Surface Glycoprotein CD6 Isoform (β = 0.16, *P* = .042) and Hepatocyte Growth Factor (β = 0.15, *P* = .049) (Supplementary Tables 7, http://links.lww.com/MD/M902).

In UC, the IVW method revealed a negative correlation between *Lachnospiraceae UCG008* and C-X-C Motif Chemokine 9 (β = −0.09, *P* = .029), and between *Ruminococcus 2* and C-C Motif Chemokine 4 (β = −0.11, *P* = .034) (Supplementary Tables 8, http://links.lww.com/MD/M903).

### 3.5. MVMR results and mediation effects

**Figure 5.**
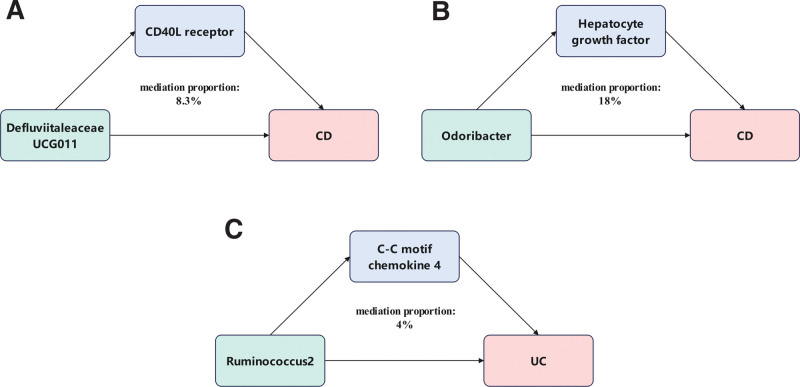
(A) The mediating effect of CD40L receptor in the causal relationship between *Defluviitaleaceae UCG011* and CD. (B) The mediating effect of Hepatocyte Growth Factor in the causal relationship between *Odoribacter* and CD. (C) The mediating effect of C-C motif chemokine 4 in the causal relationship between *Ruminococcus2* and UC. CD = Crohn disease, UC = ulcerative colitis.

Following the MVMR analysis and after adjusting for each other, in CD (Supplementary Tables 9, http://links.lww.com/MD/M904), we found that *Defluviitaleaceae UCG011* (β = 0.22, *P* = .006) and CD40L receptor (β = −0.17, *P* < .001) showed that the mediating effect of CD40L receptor was β = 0.02 with a total effect of β = 0.24, making the mediation proportion 8.3% (Fig. 5A). For *Defluviitaleaceae UCG011* (β = 0.23, *P* = .08) and C-X-C motif chemokine 9 (β = 0.37, *P* = .001), the mediating effect was β = −0.05 and the total effect was β = 0.18, but due to the opposite direction of β values, mediation was not established. *Odoribacter* (β = 0.34, *P* = .08) and T-cell surface glycoprotein CD6 isoform (β = 0.09, *P* = .010) were not significant. For *Odoribacter* (β = 0.24, *P* = .45) and Hepatocyte growth factor (β = 0.36, *P* = .03), the mediating effect of Hepatocyte growth factor was β = 0.05 with a total effect of β = 0.29, making the mediation proportion 18% (Fig. 5B).

In UC, after mutual adjustment (Supplementary Tables 10, http://links.lww.com/MD/M905), *Lachnospiraceae UCG008* (β = 0.21, *P* = .009) and C-X-C motif chemokine 9 (β = 0.25, *P* = .001) showed that the mediating effect was β = −0.02 and the total effect was β = 0.19. Due to the opposite direction of β values, mediation was not established. *Ruminococcus2* (β = −0.22, *P* = .016) and C-C motif chemokine 4 (β = 0.13, *P* < .001) showed a mediating effect of C-C motif chemokine 4 of β = −0.01 with a total effect of β = −0.23, making the mediation proportion 4% (Fig. 5C).

## 4. Discussion

Although the specific pathogeneses of CD and UC are not yet fully understood,^[32]^ increasing evidence suggests complex interactions between inflammatory responses, dysbiosis of gut microbiota structure, and intestinal barrier dysfunction, all contributing to the development of CD and UC.^[33,34]^ This study employed the MR methods, based on multiple GWAS datasets, to explore the causal relationships and potential mediators between gut microbiota and inflammatory proteins in CD and UC. Through our analysis, we identified 5 types of gut microbiota associated with CD and 6 types associated with UC, as well as 6 inflammatory proteins related to CD and 11 related to UC. Furthermore, the CD40L receptor may mediate 8.3% of *Defluviitaleaceae UCG011* effect on CD. HGF may mediate 18% of *Odoribacter* effect on CD; CCL4 may mediate 4% of *Ruminococcus2* effect on UC. Our study provides evidence of a causal relationship between gut microbiota and CD and UC, as well as the mediating effects of the CD40L receptor, HGF, and CCL4.

Our analysis indicates that *Defluviitaleaceae UCG011, Odoribacter, Parasutterella*, and *Ruminococcaceae UCG014* are associated with an increased risk of CD, while *Ruminococcaceae UCG009* is beneficial in reducing the risk of CD. Research has found that an increase in the abundance of *Odoribacter* in the gut can modulate the homeostasis of regulatory T cells through the production of butyrate, thereby exerting immunoregulatory and anti-inflammatory properties.^[35–37]^ However, this contradicts our findings; our study suggests that *Odoribacter* may promote the occurrence of CD, indicating that the role of *Odoribacter* in CD requires further exploration. *Ruminococcaceae UCG009*, as an anti-inflammatory bacterium, plays an important role in the production of SCFAs, especially in maintaining intestinal epithelial integrity and inhibiting inflammation,^[38–40]^ suggesting that *Ruminococcaceae UCG009* could reduce the risk of CD through the production of SCFAs. For UC, we discovered that *Eubacterium eligens* and *Lachnospiraceae UCG008* are associated with an increased risk of UC, while *Clostridium sensu stricto 1, Eubacterium ventriosum, Oscilibacter*, and *Ruminococcus2* are related to a decreased risk of UC. *Lachnospiraceae UCG-008* has a close association with T cells and NK cells, indicating it may also lead to UC through inflammatory responses,^[41]^ whereas *Clostridium sensu stricto 1* is crucial for intestinal health through its production of butyrate.^[42]^ Additionally, an increase in *Eubacterium ventriosum* is associated with a decrease in the inflammatory mediators IL-6 and IL-8, suggesting it may influence the development of UC by inhibiting inflammation^[43]^; *Ruminococcus2*, also a SCFA-producing bacterium, plays a vital role in maintaining internal balance and intestinal development.^[44]^

In our study on the impact of inflammatory proteins on CD and UC, in CD, we found that levels of Eotaxin-1, T-cell surface glycoprotein CD6 isoform, CXCL9, FGF21, and HGF are positively correlated with the development of CD. Levels of the CD40L receptor are negatively correlated with the development of CD. Eotaxin-1 activates eosinophils and mobilizes them to the intestinal lamina propria,^[45,46]^ participating in the initiation and progression of inflammatory responses, thus leading to intestinal tissue damage and dysfunction.^[47]^ CXCL9, as a chemokine expressed during immune responses, is associated with increased inflammation in various autoimmune diseases,^[48–51]^ indicating its possible contribution to the development of CD. In UC, our study indicates that increased levels of Axin-1, CCL20, CCL4, CXCL11, CXCL9, FGF21, IL-10RA, and IL-18 promote the development of UC. Levels of CD40L receptor, C-X-C motif chemokine 5, and TNF-beta inhibit the development of UC. CXCL11 serves as a chemotactic factor for eosinophils and various leukocytes in inflammatory conditions, playing a significant role in the recruitment of these cells to sites of inflammation,^[52]^ potentially meaning that it promotes the development of inflammation through its chemotactic activity; Meanwhile, IL-18 promotes the occurrence of colitis by affecting IL-22/STAT3 signaling and the number of goblet cells.^[53]^ Notably, although our data mainly come from European populations, previous studies have found that IL-10RA dysfunction is a major cause of very early-onset IBD (VEO-IBD) in East Asian populations, where a potential mechanism might be a novel 333-bp deletion in IL10RA.^[54]^ Mentioning this aims to highlight the importance of genetic background in IBD onset and the potential differences among various populations. Furthermore, it suggests that future research should more broadly explore the diversity of IBD pathogenesis mechanisms across different genetic backgrounds.

Despite the concentration of current research on the effection of gut microbiota and inflammatory proteins to CD and UC, the mediating role of inflammatory proteins in how gut microbiota affects the CD and UC process has rarely been explored until now. Recent research has revealed the critical role of inflammatory proteins in mediating between gut microbiota and disease. For example, *Bacteroides fragilis synthesizes polysaccharide A*, which has the capability to prevent colitis triggered by *Helicobacter hepaticus*. This is achieved by suppressing the interleukin-17 and boosting the secretion of IL-10.^[55]^ Furthermore, exposure to *commensal luminal bacteria* has been found to stimulate the production of IL-12,^[56]^ which is considered to be involved in autoimmune diseases induced by bacterial products.^[57]^ These findings emphasize the central role of inflammatory proteins in regulating the balance between gut microbiota and host health, providing us with new perspectives on studying the pathogenesis of CD and UC.

Based on current research, we explored the potential mediating role of specific inflammatory proteins between gut microbiota and CD and UC. Our mediation analysis found that inflammatory proteins are involved in the microbiota impact on CD and UC. Specifically, we found that *Defluviitaleaceae UCG011* mediates its effect on CD through the CD40L receptor. The main binding receptor for CD40L is CD40, which as a receptor of the TNF superfamily is widely expressed in immune cells, participating in various immune responses such as those involving B cells, and macrophages, as well as in non-immune cells such as endothelial cells, epithelial cells, and mesenchymal cells,^[58,59]^ suggesting that the CD40L receptor may play a crucial role in mediating immune responses through the CD40-CD40L pathway in the process of *Defluviitaleaceae UCG011* impact on CD.

Additionally, HGF, a protein that stimulates DNA synthesis in liver cells,^[60]^ enhances processes such as the formation of new blood vessels, cell movement, proliferation, invasion, development of form, embryogenesis, tissue repair, and healing of injuries through signal transduction following its binding with tyrosine-protein kinase Met (c-MET).^[61]^ Preliminary studies indicate that HGF primarily aids intestinal repair by promoting intestinal epithelial regeneration, accelerating such regeneration, stimulating cell proliferation, and preventing apoptosis to ameliorate experimental colitis,^[62,63]^ a finding that diverges from ours. Reflecting on the inconsistency with existing research, we hypothesize that in the context of CD, a complex inflammatory condition, the role of HGF may vary depending on the environment, sometimes playing differing or even opposing roles in disease progression. Specifically, the mediating effect of *Odoribacter* through HGF may involve complex interactions between the gut microbiota and the host. This includes, but is not limited to, influencing changes in the gut microenvironment, modulating immune responses, or altering intestinal barrier functions, potentially playing crucial roles in the initiation and progression of CD. Therefore, we speculate that *Odoribacter* may affect the development of CD through various signaling pathways of HGF.

In UC, *Ruminococcus2* affects UC through mediation by CCL4. CCL4, as part of the chemokine family, is primarily produced by immune cells such as macrophages and dendritic cells, playing a role in the immune system. CCL4 can attract immune cells to the site of inflammation, such as T cells, monocytes, and dendritic cells, promoting the development of immune and inflammatory responses.^[64]^ Previous Mendelian studies have also found a suggestive association between CCL4 and UC.^[23]^ Experimental studies have also found that treated UC patients have significantly lower serum CCL4 levels compared to untreated patients.^[65]^ *Ruminococcus2* not only produces SCFA but also produces secondary bile acids (SBAs). SBAs exerts its anti-inflammatory effects further through interaction with the farnesoid X receptor (FXR) in the intestine. Activation of FXR can significantly alleviate intestinal mucosal inflammation by suppression of pro-inflammatory cytokines, including IL-1 and TNF-α.^[66–68]^ We propose the hypothesis that SBAs produced by *Ruminococcus2* interacts with FXR, thereby inhibiting the release of CCL4 and thus reducing intestinal inflammatory responses. In summary, our study reveals the mediating role of inflammatory proteins between gut microbiota and the development of CD and UC, offering new perspectives for understanding the complex pathogenesis of these diseases.

The limitations of our study also need to be acknowledged. First, the analysis of MR provides a good method to validate the causality between the 2. However, the analysis of MR reflects the exposure to genes over a lifetime, rather than short-term effects. This may not reflect the benefits of short-term changes in the gut microbiota. Yet, it can still inform us of the potential direction of influence, which requires further research to verify. Moreover, our study is only on Europeans, which may have some limitations in applicability to other ethnic groups. Future research is needed to explore the impact of gut microbiota and inflammatory proteins on other ethnic groups.

## 5. Conclusion

Our study highlights the role of the gut microbiota and inflammatory proteins in regulating immune responses and their potential impact on CD and UC. The identified associations and mediating effects pave the way for various future research avenues. It emphasizes the importance of the gut-immune axis in health and disease.

## Acknowledgments

All data used in this study were sourced from publicly accessible databases. We thank all participants and investigators for contributing to those databases.

## Author contributions

**Conceptualization:** Yu-Liang Huang.

**Data curation:** Yu-Liang Huang, Jin-Min Zheng, Zheng-Yi Shi, Huan-Huan Chen.

**Formal analysis:** Yu-Liang Huang, Jin-Min Zheng, Huan-Huan Chen.

**Funding acquisition:** Jin-Min Zheng, Huan-Huan Chen.

**Investigation:** Jin-Min Zheng, Zheng-Yi Shi.

**Methodology:** Zheng-Yi Shi, Fan-Biao Kong.

**Project administration:** Xiao-Tong Wang, Fan-Biao Kong.

**Resources:** Xiao-Tong Wang, Fan-Biao Kong.

**Software:** Xiao-Tong Wang, Fan-Biao Kong.

**Supervision:** Xiao-Tong Wang, Fan-Biao Kong.

**Validation:** Xiao-Tong Wang, Fan-Biao Kong.

**Visualization:** Xiao-Tong Wang, Fan-Biao Kong.

**Writing – original draft:** Yu-Liang Huang.

**Writing – review & editing:** Jin-Min Zheng, Zheng-Yi Shi, Huan-Huan Chen, Xiao-Tong Wang, Fan-Biao Kong.

## Supplementary Material
























