# Supplementary material for: Inflammatory proteins may mediate the causal relationship between gut microbiota and inflammatory bowel disease: A mediation and multivariable Mendelian randomization study
Source: Medicine (Baltimore). 2024 Jun 21;103(25):e38551. doi: 10.1097/MD.0000000000038551 (PMC11191895; doi:10.1097/MD.0000000000038551)
Supplement: Supplementary file 8 [file medi-103-e38551-s008.docx]

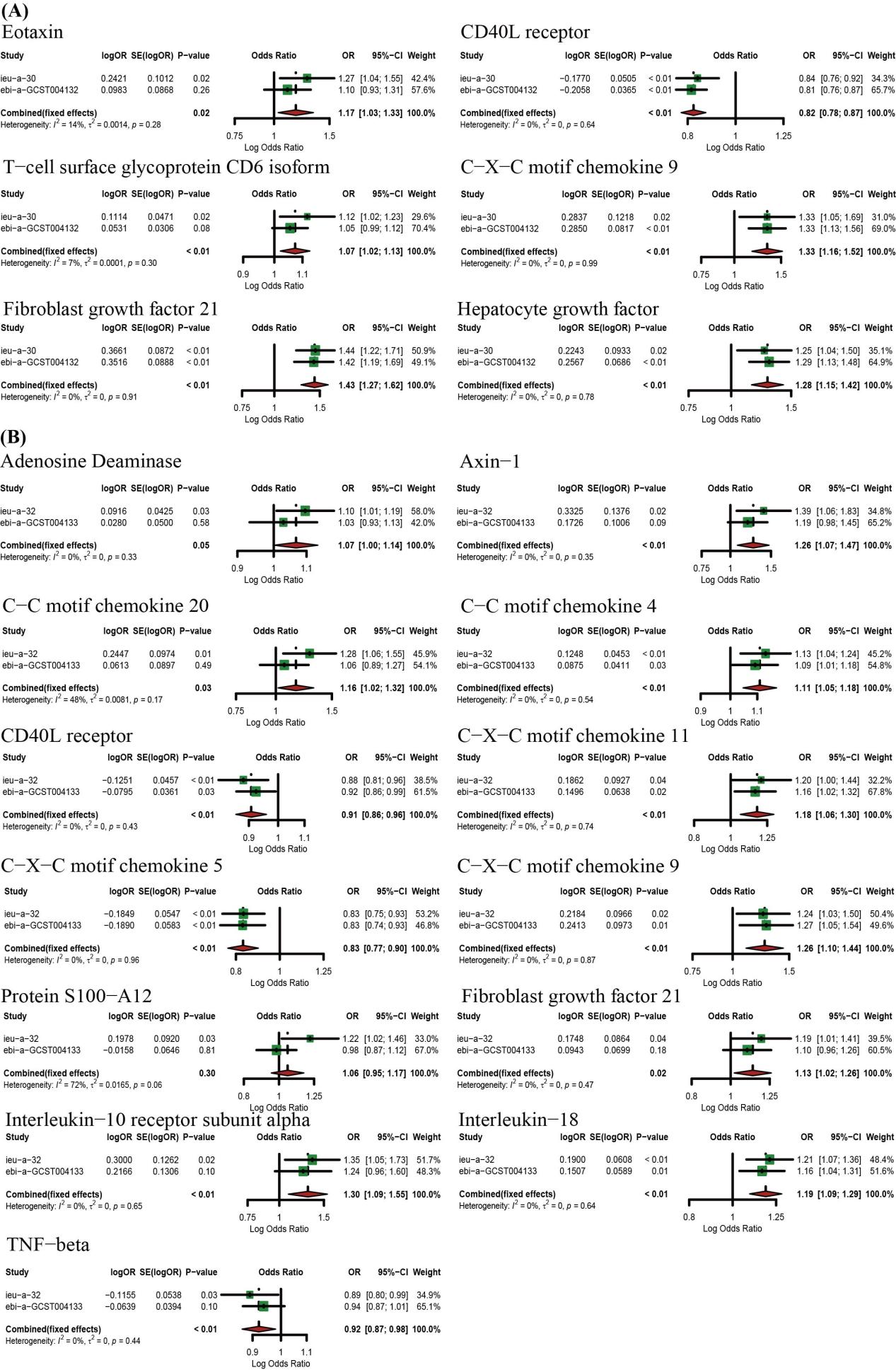


**Supplementary Figure S2 (A)**Meta-analysis of the causal association between candidate inflammatory proteins and CD;**(B)**Meta-analysis of the causal association between candidate inflammatory proteins and UC
